# Detection of *Rickettsia felis*, *Rickettsia typhi*, *Bartonella* Species and *Yersinia pestis* in Fleas (Siphonaptera) from Africa

**DOI:** 10.1371/journal.pntd.0003152

**Published:** 2014-10-09

**Authors:** Hamza Leulmi, Cristina Socolovschi, Anne Laudisoit, Gualbert Houemenou, Bernard Davoust, Idir Bitam, Didier Raoult, Philippe Parola

**Affiliations:** 1 Aix Marseille Université, URMITE, UM63, CNRS 7278, IRD 198, Inserm 1095, Marseille, France; 2 The University of Liverpool, Institute of Integrative Biology, University of Liverpool, Liverpool, United Kingdom; 3 University of Antwerp, Evolutionary Ecology, Groenenborgerlaan, Antwerp, Belgium; 4 Unité de Recherche en Zoogeographie, Universite de Liège, Sart Tilman, Belgique; 5 Université de Boumerdes Laboratoire VALCORE, Faculté des Sciences, Bourmedes, Algérie; 6 Université de Bab Ezzouar, Laboratoire d'Ecologie et Environnement, Alger, Algérie; University of California, San Diego, School of Medicine, United States of America

## Abstract

Little is known about the presence/absence and prevalence of *Rickettsia* spp, *Bartonella* spp. and *Yersinia pestis* in domestic and urban flea populations in tropical and subtropical African countries.

**Methodology/Principal findings:**

Fleas collected in Benin, the United Republic of Tanzania and the Democratic Republic of the Congo were investigated for the presence and identity of *Rickettsia* spp., *Bartonella* spp. and *Yersinia pestis* using two qPCR systems or qPCR and standard PCR. In *Xenopsylla cheopis* fleas collected from Cotonou (Benin), *Rickettsia typhi* was detected in 1% (2/199), and an uncultured *Bartonella sp*. was detected in 34.7% (69/199). In the Lushoto district (United Republic of Tanzania), *R. typhi* DNA was detected in 10% (2/20) of *Xenopsylla brasiliensis*, and *Rickettsia felis* was detected in 65% (13/20) of *Ctenocephalides felis strongylus*, 71.4% (5/7) of *Ctenocephalides canis* and 25% (5/20) of *Ctenophthalmus calceatus calceatus*. In the Democratic Republic of the Congo, *R. felis* was detected in 56.5% (13/23) of *Ct. f. felis* from Kinshasa, in 26.3% (10/38) of *Ct. f. felis* and 9% (1/11) of *Leptopsylla aethiopica aethiopica* from Ituri district and in 19.2% (5/26) of *Ct. f. strongylus* and 4.7% (1/21) of *Echidnophaga gallinacea*. *Bartonella sp*. was also detected in 36.3% (4/11) of *L. a. aethiopica*. Finally, in Ituri, *Y. pestis* DNA was detected in 3.8% (1/26) of *Ct. f. strongylus* and 10% (3/30) of *Pulex irritans* from the villages of Wanyale and Zaa.

**Conclusion:**

Most flea-borne infections are neglected diseases which should be monitored systematically in domestic rural and urban human populations to assess their epidemiological and clinical relevance. Finally, the presence of *Y. pestis* DNA in fleas captured in households was unexpected and raises a series of questions regarding the role of free fleas in the transmission of plague in rural Africa, especially in remote areas where the flea density in houses is high.

## Author Summary

Fleas are associated with many bacterial diseases such as rickettsioses, bartonelloses and plague. These diseases may be severe, and little is known about their prevalence. Accordingly, we believe that our data shed light on the problem of unexplained fevers in tropical and subtropical African areas. Using molecular tools, we surveyed and studied selected flea-borne agents, namely *Rickettsia* spp. (*R. felis* and *R. typhi*), *Bartonella* spp. and *Y. pestis*, in fleas collected in Ituri (Linga and Rethy health zone) and Kinshasa in the Democratic Republic of the Congo, the Lushoto district in the United Republic of Tanzania and in Cotonou in Benin. We found that these bacteria are present in the studied regions. *R. typhi* and an unidentified *Bartonella sp.* were detected in fleas from Cotonou (Benin). *R. felis* and *R. typhi* were also detected in the Lushoto district (United Republic of Tanzania). Finally, we detected *R. felis*, *Bartonella sp.* and *Y. pestis* in the Democratic Republic of the Congo. As these emerging zoonotic diseases have a global distribution and affect public health, the implementation of vector control strategies is urgent.

## Introduction

The importance of fleas in human and animal health is largely related to their ability to transmit agents of infectious diseases [1]. The transmission of these zoonotic agents to human occurs mainly through bites or inoculation of feces into pruritic bite lesions [2], [3]. Plague is the most notorious flea-borne disease known to man and is a reemerging public health issue mainly in Africa and South America [3]. The etiological agent of plague, *Yersinia pestis*, is a facultative gram-negative bacterium restricted nowadays to well defined endemic foci [4], [5]. In the last decade, plague reemerged in old quiescent foci of Algeria [6], the United Republic of Tanzania [7] and Libya [8] and caused remarkable bubonic and pneumonic outbreaks in known endemic foci in Madagascar [9] and in the Democratic Republic of the Congo [10]. Fleas are also associated with other bacterial diseases such as bartonelloses and rickettsioses. *Rickettsia* spp., the etiological agents of rickettsioses, are intracellular gram-negative bacteria that represent an emergent global threat, particularly in the tropics [11]. *R. felis*, an emerging pathogen, and *R. typhi*, the agent of murine typhus (MT), are the main rickettsial pathogens associated with fleas [1], belonging to the spotted fever group (SFG) [12] and typhus group rickettsiae, respectively [13]. Although these two flea-borne rickettsiae are distributed worldwide, *R. typhi* appears to be more endemic in tropical regions, coastal areas and ports, where its transmission cycles between rats (*Rattus* spp.) and oriental rat fleas (*X. cheopis*) [14]. Also, several closely related rickettsiae, referred as Rickettsia felis–like organisms (RFLO), identified in fleas and other arthropods around the world [15]. Likewise, bartonelloses are diseases caused by the fastidious, hemotropic bacteria of the genus *Bartonella*, especially in debilitated and immunocompromised individuals [16]. Importantly, the list of host species harboring *Bartonella* spp. includes a large number of mammals, mostly rodents, some of which are kept as pets [17].

An increasing number of papers have reported the occurrence of fleas and human flea-borne infections, especially in relation to wildlife and zoonotic risk. However, the identity and distribution of flea-borne agents in urban, domestic or peridomestic settings have been poorly documented in Sub-Saharan African countries such as the Democratic Republic of the Congo, the United Republic of Tanzania and Benin. Historical data about human infection with *Rickettsia* and *Bartonella* species are fragmentary, and virtually nothing is known about the current distribution of these flea-borne zoonotic agents in potential vectors and reservoir hosts in these countries.

In the Democratic Republic of the Congo, recent small-scale surveys have reported serological evidence for *Bartonella* infection in human patients [18] and molecular data in rodents [19] and fleas [20], suggesting a global underreporting at the country scale. Rickettsioses in humans are mentioned in historical reports; however, their notification remains anecdotal, and the species identification is likely erroneous. Recently though, among febrile military patients in Kisangani, Democratic Republic of the Congo, one patient tested positive in 1999, for the *R. typhi* antigen using serological tools. In addition, *R. felis* has been found to circulate in arthropod vectors in Kinshasa [21]. As a general trend, flea-borne agents in fleas are underreported, whereas in the United Republic of Tanzania, a growing number of publications confirm their presence and wide distribution in humans [22] exposed to their bites and in infested rodents [19].

In recent years, our laboratory (Unité de Recherche sur les Maladies Infectieuses et Tropicales, the WHO Collaborative Centre for Rickettsial Diseases and Other Arthropod-Borne Bacterial Diseases in Marseille, France) initiated collaboration with correspondents and universities in the United Republic of Tanzania, the Democratic Republic of the Congo and Benin.

The present survey pursued the objectives of detecting the presence and identity of *Rickettsia* spp., *Bartonella* spp. and *Y. pestis* in flea specimens collected from domestic and peridomestic areas in the Democratic Republic of the Congo, the United Republic of Tanzania and Benin within the context of entomological studies.

## Methods

### Ethical considerations

Risk assessment was submitted to and approved by the ethical committee and decision board of each institution involved in small mammals trappings, and involved informed consent of the domestic animal owners; ethical approval are available from original publications on mammal hosts on which flea were collected [19], [23], [24]. The Ethical commitee of the University of Antwerp, Belgium and the Sokoine University of Agriculture Morogoro under the project RATZOOMAN granted by the European Commission Framework 5 Programme on International Cooperation, project contract number ICA4 CT 2002 10056, approved the experiment in the South-eastern Africa.

See here technical annex: http://projects.nri.org/ratzooman/docs/technical%20annex.pdf.

### Sites of study and flea collection

**Figure 1:**
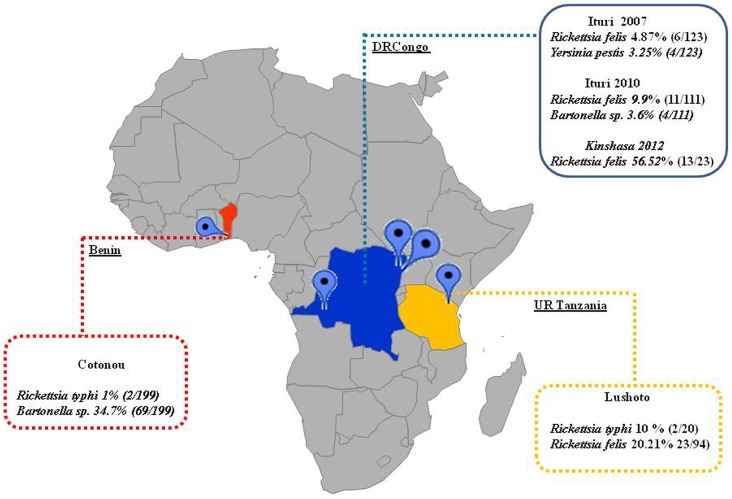
Sites of the study.

The material analyzed consisted of fleas (Siphonaptera) collected in domestic and peridomestic areas in Benin, the United Republic of Tanzania and the Democratic Republic of the Congo (Figure 1). A portion of the collected fleas was used for the present study. A convenient sample was selected according to a good representation of species, host and localities.

In 37 sites in the capital city of Benin, Cotonou (6°21′36″N; 2°26′24″E), rodent fleas were collected from rodents trapped monthly inside human residences and peridomestic areas between November 2009 to July 2010, as described previously [24]. In the United Republic of Tanzania, 17 sites in the Lushoto district (04°40′00″S 38°19′00″E) located in the Tanga Region were surveyed [23], [25]. Lushoto district is a mountainous area where plague was reported from the first time in 1981; this endemic plague focus has however been quiescent since 2004. Between May 2005 and November 2008, fleas were collected – as in Benin – from small mammals in domestic and peridomestic habitats during the dry and rainy seasons. Further details on the rodent measurements and flea collection have been published elsewhere [23], [25].

Finally, in March and April 2007, rodent fleas and free domestic fleas were collected from 4 villages (15 capture sites) in the Linga and Rethy health zones, Ituri district, Orientale Province, the Democratic Republic of the Congo; off-host fleas were collected in 4 villages during an investigation following a plague outbreak that occurred in the third trimester of 2006 [26]. Our investigation area was limited to Djalusene (2°12′10″5 N 30°88′02″7 E) and Kpandruma (2°05′90″1 N 30°88′70″4 E), which had confirmed plague patients, and Wanyale (2°10′11″8 N 30°80′60″5 E) and Zaa (2°14′03″2 N 30°85′65″9 E), which had several suspect cases but were considered control areas at the time of the study. We collected fleas in 40 houses (bedroom) in each village, for 3 nights in a row, using a kerosene lamp hung above a 45-cm diameter tray containing water as described in [27]. In April 2010 and July 2012, additional flea samples were collected from the Ituri district in Rethy village (1°50′N 29°30′E) and in Kinshasa (4°19′19″S 15°19′16″E) by means of light traps in human residences (bedroom) and rodent burrows, and flat tweezers on dogs.

All fleas collected in Benin, the United Republic of Tanzania and the Democratic Republic of the Congo were stored in 70% ethanol and identified morphologically using classical entomologic taxonomic keys. The samples were later processed in the WHO Collaborative Center for Rickettsial Diseases and Other Arthropod-Borne Bacterial Diseases, in Marseille, France.

### DNA extraction

Fleas were rinsed twice in distilled water for 10 minutes and dried on sterile filter paper; the handling was performed in a laminar flow biosafety hood. The fleas were individually crushed in sterile Eppendorf tubes, as described [28]. A total of 50 µl of DNA was extracted from one half of each flea using the QIAamp Tissue Kit (Qiagen, Hilden, Germany) by QUIAGEN-BioRobot EZ1, according to the manufacturer's instructions. The genomic DNA was stored at −20°C under sterile conditions until used as the template in PCR assays. The remaining portion of each flea was kept at −80°C for an additional control.

### Detection of *Rickettsia felis*

All samples were screened by quantitative real-time PCR (qPCR) targeting the biotin synthase (*bioB*) gene, as previously described [29]. Positive results were confirmed by another qPCR targeting a membrane phosphatase gene with primers (Rfel_phosp_MBF: 5′-GCAACCATCGGTGAAATTGA-3′ and Rfel_phosp_MBR: 5′-GCCACTGTGCTTCACAAACA-3′) and a probe (Rfel_phosp_MBP: 6FAM-CCGCTTCGTTATCCGTGGGACC-TAMRA) designed in our laboratory. The final mixture of the qPCR reaction was composed of 15 µL of mix that contained 10 µL of master mix QuantiTect Probe PCR Kit (QIAGEN, Hilden, Germany), 0.5 µL (20 pmol) of each primer, 0.5 µL (62.5 nmol) of probe, 3.5 µL of RNase DNase-free water and 5 µL of DNA extracted from fleas. qPCR was performed as follows: 15 min at 95°C, followed by 40 cycles of 1 s at 95°C, 40 s at 60°C and 40 s at 45°C, as described [29]. The negative control consisted of DNA extracted from uninfected fleas from our laboratory colony and was used for all the PCR assays in this work. The positive control was DNA extracted from a diluted strain of *R. felis* from our laboratory in Marseille. Positive results were recorded if the cycle threshold (Ct) value obtained was lower than 36 using the 2 PCR systems.

### Detection of *Rickettsia typhi*

Samples were screened by qPCR targeting a fragment of the Rpr 274P gene coding for a hypothetical protein, as described previously [30]. Positive results were confirmed by qPCR targeting the glycosyltransferase gene using a previously described Rpr 331 system [31]. qPCR was conducted using the same method as described for *R. felis* detection. The positive control was DNA extracted from a diluted strain of *R. typhi Wilmington* (ATCC VR-144) cultured in our laboratory in Marseille.

### Detection of *Bartonella* spp

DNA samples were screened by quantitative real-time PCR targeting the ITS region [32]. Positive samples with ITS primers were then confirmed by standard PCR performed with *Bartonella*-specific primers for the citrate synthase (*gltA*) gene, amplifying an approximately 334-bp fragment [33]. The positive control was *B. alsatica* strain IBS 382 (CIP 105477) DNA extracted from a strain and previously diluted to 10^−6^.

The success of PCR amplification was verified by 2% agarose gel migration. The products were purified using NucleoFast 96 PCR plates (Machery-Nagel EURL, France) as recommended by the manufacturer. The purified PCR products were sequenced with *gltA* primers using the BigDye version 1.1 cycle sequencing ready reaction mix (Applied Biosystems, Foster City, CA) with the ABI 31000 automated sequencer (Applied Biosystems). The sequences were assembled and analyzed with the ChromasPro program (version 1.5).

### Detection of *Yersinia pestis*

DNA samples were screened by qPCR targeting the plasminogen activator gene (*Pla*) [6] using primers Yper_PLA_F (5′-ATG-GAG-CTT-ATA-CCG-GAA-AC-3′) and Yper_PLA_R (5′-GCG-ATA-CTG-GCC-TGC-AAG-3′) and probe Yper_PLA _P (6- FAM-TCC-CGA–AAG-GAG-TGC-GGG-TAA-TAGG-TAMRA). Positive results were confirmed with standard PCR targeting the glpD gene, as described [34], and then sequenced using the same method used for *Bartonella* spp. sequencing. The positive control was *Y. pestis* DNA extracted from the CSUR P 100 strain, and diluted to 10^−6^.

## Results

### Benin

In Benin, 886 fleas were collected from 199 sexually mature small mammals of four species, namely, *Crocidura olivieri* (17/199, 8.5%), *Mastomys natalensis* (36/199, 18%), *Rattus norvegicus* (40/199, 20.1%) and *Rattus rattus* (109/199, 54.7%). Three flea species were collected from rodents, with the oriental rat flea *X. cheopis* being the most abundant (861/886, 97.1%), followed by *X. brasiliensis* (24/886, 2.7%) and *Ct. felis strongylus* (1/886, 0.1%). In the present study, a convenient sample of 199 *X. cheopis* (picked off *Rattus rattus*) individuals – 55.78% females and 44.2% males – were selected for an initial molecular screening (the remaining fleas were preserved for subsequent studies).

All fleas tested negative for *R. felis* and *Y. pestis*. qPCR performed for the detection of *R. typhi* revealed 2 positive *X. cheopis* (2/199, 1%), with a Ct of 32.6 and 34.5, from 2 sites (Bokossi Tokpa and Dédokpo). *Bartonella* spp. were detected in 69/199 (34.6%) of the fleas (Ct, 31.81, +/−2.97) (24≤Ct≤35) collected from all studied sites (Table 1). DNA sequence analyses of the PCR products of the gltA gene of 8 representative samples (with high Ct values) showed 100% similarity with the Uncultured *Bartonella* sp. clone Pd5700t (GenBank no. FJ851115.1, 334/334 bp) detected in *Praomys delectorum* rodents in Mbulu district, northern Tanzania [19]. More information about the Ct value and localization of each positive flea is reported in Supplementary data S1.

**Table 1:** Distribution of the positive fleas according to species and country.

Country		Fleas species	Total	Number of fleas positive to			
				*R. felis*	*R. typhi*	*Bartonella sp*	*Yersinia pestis*
BENIN		*X. cheopis*	199	-	**2**	**69**	-
UNITED REPUBLIC OF TANZANIA		*Ct. f. strongylus*	20	**13**	-	-	-
		*Ct. canis*	7	**5**	-	-	-
		*Ct. ca. calceatus*	20	**5**	-	-	-
		*X. brasiliensis*	20	-	**2**	-	-
		*P. irritans*	20	-	-	-	-
		*N. incisus*	7	-	-	-	-
DEMOCRATIC REPUBLIC OF THE CONGO	**ITURI (2007)**	*Ct. f. strongylus*	26	-	-	-	**1**
		*E. gallinacea*	21	-	-	-	-
		*P. irritans*	30	-	-	-	**3**
		*X. brasiliensis*	19	-	-	-	-
		*L. ae. aethiopica*	1	-	-	-	-
		*T. penetrans*	26	-	-	-	-
	**ITURI (2010)**	*Ct. f. felis*	38	**10**	-	-	-
		*X. cheopis*	62	-	-	-	-
		*L. ae. aethiopica*	11	**1**	-	**4**	-
	**KINSHASA**	*Ct. f. felis*	23	**13**	-	-	-

### United Republic of Tanzania

A total of 3821 fleas (rodent fleas and free-roaming fleas present in the environment) were collected from localities of the Lushoto district (United Republic of Tanzania) and were distributed into 23 species. A total of 94 fleas belonging to six common species were screened (Supplementary data S2) (20 *Ct. f. strongylus*, 7 *Ct. canis*, 20 *Ctenophthalmus calceatus calceatus*, 20 *X. brasiliensis*, 20 *Pulex irritans* and 7 *Nosopsyllus incisus*. All tested fleas were negative for *Y. pestis* and *Bartonella* spp. DNA. However, *R. typhi* DNA was detected in 10% (2/20) of *X. brasiliensis* collected from 2 villages (Magamba and Manolo). *R. felis* DNA was also detected in 20.2% (23/94) of analyzed fleas, including 65% (13/20) of *Ct. f. strongylus*, 71.4% (5/7) of *Ct. canis* and 25% (5/20) of *Ct. ca. calceatus*.

### Democratic Republic of the Congo

In 2007, in the Linga and Rethy health zones, Ituri district, 1190 fleas captured in households, belonging to 6 species (394 *P. irritans*, 153 *Tunga penetrans*, 280 *Ct. f. strongylus*, 89 *Echidnophaga gallinacea*, 88 *L. a. aethiopica* and 186 *X. brasiliensis*). A total of 123 fleas were conveniently selected for this work (Supplementary data S3). qPCR for *R. typhi* and *Bartonella* spp. was negative for all 123 fleas; however, 4.8% (6/123), namely 19.2% (5/26) of *Ct. f. strongylus* and 4.7% (1/21) of *E. gallinacea*, contained *R. felis* DNA (Table 1).

*Y. pestis* DNA was detected in 3.8% (1/26) of *Ct. f. strongylus* and 10% (3/30) of *P. irritans* from 2 villages (Wanyale and Zaa). DNA sequence analyses of the PCR products targeting the glpD gene showed 100% similarity with *Yersinia pestis Angola* isolated from Angola (GenBank accession no. CP000901.1, 321/333 bp).

In 2010, 111 fleas, belonging to 3 species, were collected in the same district, namely, *X. cheopis* (62/111, 55.8%), *Ct. f. felis* (38/111, 34.2%) and *L. a. aethiopica* (11/111, 9.9%) (Supplementary data S4). qPCR for *R. typhi* and *Y. pestis* detection was negative for all fleas (Table 1); however, 9.9% (11/111) of two flea species (*Ct. f. felis* and *L. a. aethiopica*) were positive for *R. felis*. A total of 10 *Ct. f. felis* from 38 tested (26.3%) and one of 11 *L. a. aethiopica* (9%) contained *R. felis*. *Bartonella* spp DNA was detected in 3.6% (4/111) of fleas, with 36.36% (4/11) from only *L. a. aethiopica*. Sequencing of the *gltA* gene fragment from these four *Bartonella*-positive samples showed 100% similarity with *Bartonella sp.* MN-ga6 (GenBank no. AJ583126.1, 320/334 bp) detected in fleas collected in South Africa.

Finally, in 2012, from the fleas collected in Kinshasa (Table 1), 56.5% (13/23) of *Ct. f. felis* collected from 3 dogs was positive for *R. felis* but negative for *R. typhi, Bartonella* spp. and *Y. pestis* by qPCR.

## Discussion

We report the first direct evidence of *R. typhi* and *Bartonella* sp. in *X. cheopis* fleas in Benin (Cotonou). In Lushoto (United Republic of Tanzania), we detected for the first time the presence of *R. typhi* DNA in *X. brasiliensis* and *R. felis* DNA in *Ct. f. strongylus*, *Ct. canis* and *Ct. ca. calceatus*. Finally, in the Democratic Republic of the Congo, we confirmed the presence of *R. felis* in *Ct. felis* in Kinshasa and for the first time report the presence of *R. felis* and *Bartonella* DNA in *L. a. aethiopica* and, most importantly *Y. pestis* DNA in *P. irritans* and *Ct. felis* from Wanyale and Zaa villages in the Rethy health zone.

The robustness of our results and the detection of these pathogens in fleas on rodents are supported by the use of a validated method of real-time PCR and subsequent sequencing. The validity of the data that we report is based on strict laboratory procedures and controls that are commonly used in the WHO Center for Rickettsial Diseases, including rigorous positive and negative controls to validate the test. Each positive qPCR result was confirmed by another specific qPCR or confirmed with a successful DNA amplification and sequencing.

*R. typhi* was detected in *X. cheopis* collected from *Rattus rattus* in Bokossi Tokpa and Dédokpo sites (Cotonou, Benin) and in *X. brasiliensis* from the United Republic of Tanzania. *X. cheopis* is the primary vector of *R. typhi*, the etiological agent of murine typhus (MT), in most locations around the world, and *X. brasiliensis* appears to be an effective vector under experimental conditions [3]. MT is most often a relatively mild disease; yet *R. typhi* can cause acute febrile illness and death [35]. The diagnosis of MT may be missed or underreported due to its non-specific symptoms or the absence of epidemiological criteria [36], [37] because laboratory tests and validated methods of diagnosis must be performed to confirm the diagnosis [30]. Before our study, *R. typhi* was never detected in Benin, and it is rarely directly reported in vectors and patients in Africa, specifically in sub-Saharan Africa. *R. typhi* in African fleas was only detected in *X. cheopis* fleas collected in Algeria [38]. Additionally, *R. typhi* has been reported in patients using serological methods in African countries [30]. Cases have been documented in international travelers returning from Tunisia, Morocco, Ivory Coast, Central African Republic, Madagascar, Reunion and Chad [30]. In the United Republic of Tanzania, a seroprevalence study among pregnant women from the port city of Dar es Salaam found a prevalence of 28% [39] and 0.5 to 9.3% in the town of Moshi and the Mbeya region, respectively [22], [40].

*R. felis* is an emergent agent of infectious disease in humans, and this agent of spotted fever is known to be maintained in cat fleas (*Ct. felis*) [41], [42]. To date, 12 species of fleas, 8 species of ticks and 3 species of mites have been found to be infected with *R. felis* [42]. This Rickettsiae has also recently been detected in several mosquito species in sub-Saharan Africa [29], [43], [44]. Interestingly, the *R. felis* genogroup seems large with recent organisms or genotypes related as *R. felis* like organisms (RFLO). Our 2 qPCR were specifically designed to amplify *R. felis* type strain (URRWXCal2). However, the biotin synthase and membrane phosphatase gene sequences of many RFLO are not known. We however know that at least our qPCR system targeting the biotin synthase (*bioB*) gene do not amplify some RFLO such as Rickettsia sp. RF2125 and Rickettsia sp. SGL01. Recently, a new qPCR assay has been proposed to address this issue by providing new qPCR primers and probe to specifically amplify *R. felis* OmpB gene fragments [15]. The clinical features of *R. felis* may include fever, fatigue, headache, generalized maculopapular rash and inoculation eschar(s) [42]. *R. felis* seems to be a frequent agent of unknown fever in Sub-Saharan Africa [44]. We detected *R. felis* in 5 species of fleas (*Ct. f. strongylus, Ct. canis, Ct. ca. calceatus*, *L. a. aethiopica* and *E. gallinacea*); some from the United Republic of Tanzania (Lushoto district), and other from the Democratic Republic of the Congo (Ituri District). *R. felis* had already been detected in the Ituri district [25], but not in *E. gallinacea*, the fowl flea, and has been previously shown to circulate in arthropod vectors (*Ctenocephalides felis*) in Kinshasa, the capital city of the country [21]. *E. gallinacea* is usually found on poultry, and can occurs on rodents (*Rattus* spp.) foraging in fowl shelters around houses [45]. While chicken DNA has been found in blood meal of fleas collected on rodents in the same area [46] other *Rickettsia* spp. antibodies have been found in poultry in Brazil [47], whether or not *R. felis* and *R. typhi* infects poultry or if poultry can act as a source of infection to human is unknown. Furthermore, no data on the potential vertical transmission of *R. felis* in *E. gallinacea*, or on the vectorial transmission of *R. felis* by *E. gallinacea* males (females are semi-sessile) between rodents and birds, are available. The questions raised by the findings of the present study in relation to *Rickettsia* in fleas are of real epidemiological significance and should be further investigated.

Molecular evidence of *Bartonella* sp. in fleas from the Democratic Republic of the Congo is supported by a recent serological survey in human patients in the Ituri who tested seropositive for *B. henselae, B. quintana* or *B. clarridgeiae* [18]. Gundi and collaborators also found that local rodents harbor *Bartonella* spp. closely related to *B. elizabethae* or *B. tribocorum* which shows that a wide variety of *Bartonella* species is present in the country, and differ according to host [19]. Bitam and collaborators [48] report that *B. elizabethae*, which causes endocarditis, and *B. tribocorum* are usually known to be transmitted by *X. cheopis* fleas. However, while in our study, we detected an Uncultured *Bartonella* sp., clone Pd5700t (GenBank no. FJ851115.1) in *X. cheopis* of Benin, we also detected *Bartonella* sp. MN-ga6 (GenBank no. AJ583126.1) in *L. a. aethiopica*, from Ituri. This *Bartonella sp.* had been previously found in the Democratic Republic of the Congo and the United Republic of Tanzania in rodents [19].

The detection of *Y. pestis* DNA in fleas collected in villages and houses where no current human plague cases had been reported for the last 6 months is puzzling. About 80 species and subspecies of Siphonaptera are known to be carriers and potentially vector of *Y. pestis* [49], via various transmission mechanisms [50]; in particular in fleas from the genus *Xenopsylla* (*X. cheopis*), which played a major role in historical plague pandemics [9]. In the present survey, DNA of *Y. pestis* was detected in the human flea, *P. irritans*, and the cat flea *Ct. felis* in a well known endemic focus of the Democratic Republic of the Congo [51]. In 2006, in the Rethy and Linga health zone more than 600 human cases were reported [52], which triggered the entomological investigation reported previously [25] and the collection of fleas analyzed herein. This survey occurred 6 months after the end of the epidemics, and at the time of the flea sampling, no confirmed human plague cases were reported to the Health centre of the villages (Zaa and Wanyale) or Rethy general Hospital. Several hypotheses can be proposed to explain this finding. A first hypothesis is that infected fleas from rodents, dogs or cats could have been imported in the infested houses, did not bite people and as such no human cases occurred, at the time of collection. A second hypothesis is that infected fleas containing *Y. pestis* DNA remained infected and alive without biting any potential host or that no human cases were reported to the health authorities which are unlikely due to the recent outbreak and constant surveillance. Other options are that *Y. pestis* DNA is reminiscent in the flea but the bacterium is either dead (degraded DNA) but the targeted sequences (gene fragment and gene flanking regions are still complete) or alive but in a quiescent form or VBNC state, possibly controlled by epigenetic mechanisms causing virulence gene repression. The human flea (*P. irritans*) may play an important role in spreading plague via human-to-human transmission as suggested in Lushoto district [27] and could possibly harbor *Y. pestis* without transmission for several months. Unfortunately no fleas were cultured in the field and the viability of the strain detected cannot be proven, but this finding calls for more research at times post outbreaks in order to answer this question. Similarly, cat fleas could play such a role both in northwest Uganda [53] and in Democratic Republic of the Congo (Laudisoit and al 2014, unpublished data), where *C. felis* spp. is the most common flea species collected in the domestic environment above a given altitude threshold.

In conclusion, we widened knowledge of the repertoire of flea-borne bacteria present in Sub-Saharan Africa. In our study, we also illustrate the role of fleas in the entomological survey of vector -borne disease, which allow clinicians to confirm the etiological cause for some of the unknown cause of fever in African patients. Future studies on rickettsioses, bartonelloses and other vector-borne diseases should be performed to assess their epidemiological and clinical relevance in tropical and subtropical areas, to estimate the real prevalence and to allow the establishment of antivectorial control plans.

## Supporting Information

Data S1 Detection of flea borne diseases in Benin.
(XLS)

Data S2 Detection of flea borne diseases in United Republic of Tanzania.
(XLSX)

Data S3 Detection of flea borne diseases in Ituri, Democratic Republic of the Congo.
(XLSX)

Data S4 Detection of flea borne diseases in Kinshasa, Democratic Republic of the Congo.
(XLSX)
